# Improving utilization of and retention in PMTCT services: Can behavioral economics help?

**DOI:** 10.1186/1472-6963-13-406

**Published:** 2013-10-10

**Authors:** Nicholas Kenji Taylor, Alison M Buttenheim

**Affiliations:** 1 Perelman School of Medicine at the University of Pennsylvania, 295 John Morgan Building, 3620 Hamilton Walk, Philadelphia, PA 19104, USA; 2 University of Pennsylvania School of Nursing, Room 235L Fagin Hall, 418 Curie Blvd, Philadelphia, Pennsylvania 19104, USA

**Keywords:** Behavioral economics, Developing countries, Health behavior, Health promotion, HIV infections, Infectious disease transmission, Vertical, Maternal health services, Medication adherence, Patient acceptance of health care

## Abstract

**Background:**

The most recent strategic call to action of the World Health Organization sets the elimination of pediatric HIV as a goal. While recent efforts have focused on building infrastructure and ensuring access to high-quality treatment, we must now turn our focus to the behavior change needed to eliminate vertical transmission. We make the case for the application of concepts from the field of behavioral economics to prevention of mother-to-child transmission (PMTCT) programs to more effectively address demand-side issues of uptake and retention.

**Discussion:**

We introduce five concepts from the field of behavioral economics and discuss their application to PMTCT programs: 1) Mentor mothers who come from similar circumstances as PMTCT patients can serve as *social references* who provide *temporally salient* modeling of utilization of services and adherence to treatment. 2) Economic incentives, like cell phone minutes or food vouchers, that reward adherence to PMTCT protocols leverage *present bias*, the observation that people are generally biased toward immediate versus future awards. 3) *Default bias*, our preference for the default option, is already being used in many countries in the form of opt-out testing, and could be expanded to all PMTCT programs. 4) We are hardwired to avoid loss more than to pursue an equivalent gain. PMTCT programs can take advantage of *loss aversion* through the use of commitment contracts that incentivize mothers to return to the clinic in order to avoid both reputational and financial loss.

**Summary:**

Eliminating vertical transmission of HIV is an ambitious goal. To close the remaining gap, innovations are needed to address demand for PMTCT services. Behavioral economics offers a set of tools that can be engineered into PMTCT programs to increase uptake and improve retention with minimal investment.

## Background

The most recent strategic call to action of the WHO sets the elimination of pediatric HIV as agoal [1]. While the past decade of research and field implementation has focused on building infrastructure and ensuring that parents have access to HIV care and treatment, additional attention must be paid to the behavior change required to eliminate vertical transmission. The goal of this debate is to introduce a set of principles from the field of behavioral economics that could be engineered into interventions to improve utilization of services and retention of mothers through high quality prevention of mother-to-child transmission (PMTCT) programs.

The prevention of mother-to-child transmission of HIV consists of four prongs: prevention of HIV among women of reproductive age, prevention of unintended pregnancies among women living with HIV, prevention of vertical transmission, and treatment [2]. PMTCT efforts over the past decade have focused on developing the proper clinical protocols to prevent vertical transmission, delivering those interventions through health care facilities, and evaluating the PMTCT health services infrastructure [3-9]. This *supply-side* approach to PMTCT in the developed world has certainly been successful, with vertical transmission rates reduced from 35% to less than 2% between the early 1990’s to present day [8]. In low-income countries, on the other hand, estimates of vertical transmission range from 15% to 40% [10].

The PMTCT cascade comprises 18 months of care from the initial antenatal visit and HIV testing through ARV treatment, intrapartum care, infant testing, infant feeding education and infant/mother treatment [11]. Results from mathematical models of the PMTCT cascade conclude that in order to reduce the number of infants infected by HIV and ensure mothers receive life-saving interventions, each step in the PMTCT cascade must be delivered (and utilized) with greater than 90% reliability [12,13]. However, recent estimates of retention of HIV-positive pregnant mothers through the full PMTCT cascade (including antenatal, intrapartum, and postpartum care) are inadequate for elimination of transmission. Based on data from the Elizabeth Glaser Pediatric AIDS Foundation, of a 100 pregnant women that attend antenatal clinic, 92 will be counseled, 77 will be tested for HIV and 69 will receive test results [14]. These numbers fall far short of the 90% retention rate necessary at each step to reduce transmission rates.

In order to address the shortcomings of current PMTCT programs, the WHO outlined seven strategic directions that are aimed at addressing the *supply-side* of the PMTCT equation, particularly focused on addressing areas such as technical guidance, integration and coordination within health care systems, and measurement of program impact on vertical transmission [1]. Undoubtedly, many structural barriers to accessing care still exist, such as transportation to clinics, lack of treatment supplies, long wait times and costly appointments [15,16]. The implicit assumption, however, in focusing on infrastructure building through this strategic direction is, “If you build it, they will come.” However, we know from a variety of health fields, from immunizations to blood pressure screenings, access to services does not necessarily mean people will use those services [17,18]. PMTCT services are no different. Even in well-resourced settings such as urban Vietnam, researchers found that, women were still not receiving counseling, were not opting in to ARV prophylaxis and were choosing not to follow feeding guidelines [19].

Recent research has identified many social and behavioral correlates of failure to access existing PMTCT services or to adhere to treatment protocols, including HIV-related stigma, exclusive breast-feeding stigma, lack of partner support and negative attitudes toward health workers [19-21]. Non-participation in PMTCT is clearly not solely related to “bad” choices by mothers– the struggle for more *supply* of quality PMTCT resources in developing countries must continue. At the same time, however, attention must now include the *demand* side of the PMTCT equation: How do we motivate HIV-positive pregnant women to utilize available PMTCT services, and to initiate and adhere to treatment protocols when the resources are available to them [1]? Researchers and policy-makers must consider the behavioral and social correlates of failure to access existing PMTCT services.

Behavioral economics, a field that builds heavily on findings from psychology, economics and finance, recognizes the inherent complexity of human decision-making and the significant influence of community, culture, and context at the moment of decision-making in everyday health decisions [22]. Interventions based on behavioral economic principles have been shown to be successful in smoking cessation, weight loss, medication adherence and maintenance of sobriety [23-27]. By understanding the many drivers behind health-related decisions, behavioral economics is one tool that can help us better address the demand-side of the PMTCT equation (i.e., increase the number of mothers who receive HIV testing, who adhere to medication recommendations and who return for visits).

Below we introduce five behavioral economics principles and suggest specific ways in which intervention workers in under-resourced settings or policymakers may apply these concepts to PMTCT interventions to address the utilization and retention challenges that currently impede progress towards the goal of eliminating mother-to-child transmission of HIV.

## Discussion

### Social references and temporal salience

Imagine you see ten people ahead of you in the checkout line at the grocery store each make a $1 donation to a local charity. What is the probability that you will make a similar donation when it is your turn? If the people ahead of you in line are all close friends, we would expect the probability to be higher than if you don’t know the other donors. The peers ahead of you in line modeling the charitable behavior are your “social references.” Generally, people judge their well-being relative to a reference point rather than in absolutes [28]. As Nolan et. al demonstrated in their work with reducing energy consumption, the greatest reductions came when customers knew the usage of their neighbors as opposed to receiving general information about environmental benefits or personal cost savings [29].

In a PMTCT pilot study that provided HIV-positive mentors to pregnant women, medical follow-up, coping skills and HIV knowledge scores all improved [30]. Mentor mothers clearly served as social reference points for the HIV-positive women. In contrast to a doctor or other health professional with whom many patients may not personally relate to, mentor mothers often come from the same circumstance, background, and even neighborhood as the clients they serve. Mentor mothers have modeled seeking HIV treatment, disclosing their HIV status to their family, and taking steps to keep their baby HIV-free.

Furthermore, the advice of mentor mothers at each clinic visit has *temporal salience* with pregnant women currently going through the PMTCT cascade. *Temporal salience*, also called *recency bias,* captures the idea that individuals apply greater weight in decision-making to information that is closest in time to the decision point [31]. Because the advice from mentor mothers is often coming immediately before a particular decision, the new information for the PMTCT patient will play more heavily in their decision-making process.

How can such concepts be applied in PMTCT settings on the ground? The use of mentor mothers can be leveraged at decision points that require discrete actions in time. For example, the decision to discuss family planning, to get an HIV test (or not refuse an HIV test), receive ARV prophylaxis during birth or have infant HIV testing are parts of the cascade where the counseling of mentor mothers just prior to the particular decision point are best suited to shape behavior. Instead of addressing breastfeeding behaviors and plans for contraception after pregnancy in only one initial intake visit, mothers can address these topics when they are most temporally salient to that particular decision point, much like the model mothers2mothers utilizes in Cape Town, South Africa. Furthermore, intervention workers may adapt training materials available through online services such as the WHO and HHS/CDC PMTCT Generic Training Package that are culturally relevant. Materials may be made more culturally relevant by eliciting suggestions from community members or using educational material delivered by an HIV-positive mother from the target community.

While it may not be feasible for mentor mothers to serve as social reference points in every clinical setting, policy makers should implement and support programming where social references are utilized to deliver temporally salient services and information. Traditional birthing attendants, for example, may serve as social reference points for HIV-positive women delivering babies outside the hospital setting in sub-Saharan Africa. Not only can traditional birthing attendants reach mothers missed in traditional outpatient clinics, they can also use their close relationship with women to improve uptake of and retention through PMTCT [32,33].

### Present bias

In an experimental study, a large US company offered financial incentives to randomly selected employees to encourage smoking cessation. Compared to the control group, the incentivized group had significantly higher rates of cessation [34]. Regular, immediate financial incentives were hypothesized to work in this case in part because people are generally biased toward immediate awards versus future awards, known as present bias or hyperbolic discounting [35]. The longer term benefits of quitting smoking, losing weight, or saving for retirement are usually more heavily discounted in favor of the immediate gratification of a cigarette, a milkshake or a night out on the town. Financial and non-financial incentives may provide a means to increase the probability of doing a hard-to-achieve behavior by making the future, uncertain, and intangible rewards of the behavior more concrete and immediate.

Present bias and hyperbolic discounting can be employed to increase utilization and retention at various stages of the PMTCT cascade. Within the PMTCT cascade, behaviors can be defined as “simple” and “complex” [36]. A simple health behavior is accomplished in one shot, such as agreeing to an HIV test or taking a particular medication in the doctor’s office. Complex health behaviors, on the other hand, require sustained behavior over a longer period of time, such as using family planning methods, breast feeding or regularly attending at appointments. One study conducted examined the role of incentives in sustained weight loss, a complex behavior. Individuals who were financially incentivized lost more weight over a sustained period of 8 months; however, participants regained the weight by week 4 post-intervention [23]. Findings such as these suggest complex behaviors are not as well suited for economic incentives. Shaping breast feeding habits or family planning practices may be less suited to leverage present bias through financial incentives.

On the other hand, simple health behaviors may be better suited for financial incentives. One randomized control trial in 2005 sent low-income uninsured US women a letter offering a free mammogram if they called a toll-free number. Some women also received a $10 financial incentive. The mammogram rates were four times higher among the group of women who received the financial incentive [37]. A similar offer may be applied in low-income countries to increase the uptake of PMTCT services. Women may be offered an economic incentive in the form of cell phone minutes or food vouchers for returning to clinic at critical points in care such as for HIV or CD4 count testing. A conditional cash transfer scheme in India to incentivize women to give birth in a health facility was shown to increase antenatal care and in-facility births. Furthermore, the incentive scheme was associated with a 3.7 reduction in perinatal deaths per 1000 pregnancies and a reduction of 2.3 neonatal deaths per 1000 live births. While the findings of cash transfer schemes or fee exemptions require further study and careful consideration of ethical issues, they demonstrate the potential for leveraging *present bias* in increasing uptake of and retention through PMTCT in a variety of under-resourced settings [38-40].

Practitioners and policy makers may also apply this concept to bolster a particularly crucial part of PMTCT: male attendance. Both antenatal attendance and HIV testing by men are associated with decreased infant HIV infection and increased HIV-free survival [20,41,42]. Furthermore, lack of male partner attendance remains a negative predictor for adherence throughout the PMTCT cascade [43,44]. However, studies have shown only 16% of women will be accompanied by their male partners. Cell phone minutes or vouchers may be additional levers of present bias that have the potential to increase male attendance by offering males an additional incentive to be at the first appointment or be present at crucial steps in the process. Programs that are bolstering male involvement in PMTCT may further incentivize men to become involved by, for example, providing work vouchers for the time lost in attending clinic visits [45].

The application of *present bias* through the use of financial incentives is not limited to a mother’s decisions in PMTCT. To address the supply side problems of access to maternal health services in Uganda, which included geographical inaccessibility, lack of transport, and financial burdens, women were given vouchers for motorcycle transport to clinic appointments and vouchers for medical providers. Initial data showed an increase in antenatal, delivery and postnatal care as well as an increase in the number of safe deliveries in the intervention group from <200 deliveries/month to over 500 deliveries/month. An additional benefit was that vouchers could then be used by providers to purchase new clinic supplies [46].

### Default bias

When you obtain or renew a driver’s license in the US, you have the option to check a box to consent to organ donation. In many other countries, on the other hand, consent is assumed and citizens are automatically enrolled as donors unless they decide to opt out. Researchers have found that even after accounting for other cultural, legal, and political differences, organ donation is 25-30% higher in countries with an opt-out system [47]. The principle behind this observation is *default bias* – our preference for the status quo or default option [48].

In 2007 the WHO issued recommendations to shift from client-initiated HIV testing and counseling (opt-in) to provider-initiated testing (opt-out) in HIV epidemics in light of mounting evidence of limited uptake of client-initiated testing for multifactorial reasons [49]. In provider-initiated HIV testing and counseling, a health provider would specifically recommend an HIV test to patients at antenatal clinic, and unless the patient declined, an HIV test would automatically be performed. Several countries where HIV is an epidemic – such as Botswana, Malawi, Uganda, South Africa and Zambia – have already made provider-initiated testing and counseling a part of country policy [50]. However, countries like China, Cambodia, India, Thailand, Kenya and Ethiopia have not yet adopted opt-out testing as a part of national policy [50].

Status quo bias predicts that an opt-out counseling and testing program should increase testing rates. Research from the field supports this hypothesis [51-54]. For example, initiation of routine HIV testing in Botswana increased testing rates from 40 per 1000 persons to 104 per 1000 persons [55]. Despite provider-initiated programs, retention rates throughout the entire PMTCT cascade remain a problem. In a rural district hospital of Malawi, 90% of mothers attending antenatal clinic accepted provider-initiated HIV testing and counseling but less than 20% of the HIV-positive mothers made it to the 6 month post-natal visit [56]. Opt-out HIV testing of newborn babies or nevirapine prophylaxis at birth may therefore be additional opportunities for researchers and policymakers to consider in order to catch women who may have been initially missed in an opt-in situation.

*Default bias* may also be leveraged at a country level to enhance the counseling on breastfeeding practices of HIV-positive mothers either on or off ARV prophylaxis as outlined by the WHO [8]. In 2010 the World Health Organization released new guidelines that recommended countries choose one infant-feeding strategy: breastfeeding with ARVs or avoidance of all breastfeeding. Prior to these recommendations, health workers individually counseled HIV-infected mothers about various feeding options and left it to mothers to decide [57]. By recommending only one default option based on local prevalence, cultural norms and government priorities, providers can focus on helping mothers feed their baby based on a unified national policy guideline and seek ARV treatment according to those guidelines. Mothers, on the other hand, are relieved of the stress that comes when faced with these difficult decisions, but are certainly supported in whatever decision they choose [58-60]. Countries continue to grapple with several challenges of implementing a default option that is consistent across all PMTCT programs, particularly that the communicating the message clearly to mothers, health care workers and policy makers [61]. While data are still scarce on the impact of implementing a default option since 2010, behavioral economics predicts that the application of *default bias* may move the needle toward reducing vertical transmission of HIV through breastfeeding.

### Loss aversion

Imagine a car salesman offers you two payment options: Option A has a 10% down payment requirement and Option B covers 90% of the cost of the car. Most people would choose Option B even though they carry the same risks and rewards. As humans, we are hardwired to avoid loss (down payment) more than to pursue an equivalent gain (loan amount), a concept known as *loss aversion*. The effect of loss aversion on decision-making has been demonstrated in situations ranging from healthcare to financial markets [62,63]. *Loss aversion* makes the threat of a loss a more powerful incentive driving behavior than the actual promise of a gain.

How can this concept be applied to PMTCT? A small savings account may be set up for families at the initial antenatal visit that can only be accessed once the 18 month infant PCR test has been completed. Each time the mother and father return for their regular appointments, money (or some monetary equivalent such as food vouchers) will be deposited into the account. If the families do not return, miss appointments, or are unaccompanied by the husband, money will be deducted from the account or they may default on eligibility to receive the entire amount. As people are more apt to avoid loss, families may feel compelled to return for follow-up appointments.

## Summary

Others have begun to look at the application of behavioral economics-based interventions in managing disease in both high-, middle- and low-income countries, many of which have been successful in impacting behavior [64-67]. However, no analysis exists of interventions applied to prevention-of-mother-to-child transmission. While the past decade of research and field implementation has been focused on building infrastructure and ensuring parents have fair access to HIV care and treatment because real barriers to care still exist, we must begin to think more critically about how to promote long-term health behaviors that will eliminate vertical transmission of HIV.

Behavioral economic principles have helped elucidate what drives behavior. These same principles have been applied in many other areas of health, such as smoking cessation, diabetes control and organ donation [25,68,69]. We make the case for their application in PMTCT to address demand-side challenges of uptake and retention as outlined in Table 1.

**Table 1:** Application of behavioral economic principles to PMTCT

**Behavioral economic concept**	**Application to PMTCT**
Social references and temporal salience	mentor mothers, support groups, culturally-relevant educational materials utilizing community members, traditional birthing attendants
Present bias	conditional cash transfers, vouchers, cell phone minutes, fee exemptions
Default bias	opt-out HIV testing and treatment, infant-feeding policy
Loss aversion	savings accounts or incremental deposits tied to PMTCT visits

Many of the investigations relating to health behavior based on behavioral economics principles have been implemented in high-income populations, while much of the disease burden of HIV occurs in low-income countries. Furthermore, behavioral economic-based interventions have not been broadly applied to PMTCT. It remains to be seen how generalizable these principles are to the primary target population: low-income, HIV-positive mothers and their families. However, a growing body of research behind the barriers and facilitators of PMTCT utilization can point us in the right direction of designing meaningful interventions. Often cited reasons of low levels of retention along the PMTCT cascade include impaired physical access, lack of PMTCT knowledge, stigma associated with HIV and lack of partner support, all of which naturally prevent parents from seeking appropriate services [70-73]. Though behavioral interventions may not be able to address physical issues of the geographic distribution of services, certainly behavioral economic principles can be applied to address behavioral barriers relating to stigma, education and partner support.

Behavioral economics provides a toolkit for policy makers and health workers. Interventions can be designed that leverage multiple behavioral economics levers. Commitment devices are one such example. Commitment devices have been shown to be powerful behavior-change mechanisms that take advantage of multiple behavioral economics principles for hard-to-achieve behaviors. For example, Stickk.com provides an online forum where users set a goal, set the stakes (both financial and reputational), invite referees to monitor achievement and invite supporters to track their progress. These contracts leverage concepts of *social reference* (referees), *present bias* (incentives), and *loss aversion* (financial and reputational stakes) [74]. In the context of PMTCT, when mothers and fathers initially enter the PMTCT cascade through their first antenatal visit, PMTCT programs may ask parents to sign a commitment contract that puts into writing the pledge of the infants’ parents to follow through with treatment. Each subsequent visit is then an opportunity to reinforce the commitment contract with parents and remind them of next steps in the process. The contracts may be further modified to leverage the concept of *present bias* by adding financial incentives. Within the contract, for example, parents may agree to return for particular critical visits in the PMTCT cascade while the government agrees to reward those behaviors through food vouchers or cell phone minutes, further nudging behavior to prevent mother-to-child transmission of HIV. Under an incremental incentives scheme that deposits monetary value into an inaccessible account, mothers may then be more inclined to come and return for services for antenatal counseling and testing in order to avoid losing money that is effectively set aside for them. Alternatively, families may be promised a fixed amount from which fines are deducted if certain milestones are not met to accentuate the loss.

The feasibility and acceptability of behavioral economics interventions remains to be seen. Of particular concern is the possible effect financial incentives may have on the motivations underlying altruistic human behaviors such as infant care [75]. Health officials and clinicians are understandably concerned about incentives “crowding out” intrinsic motivation, eventually reducing desired behavior when incentives are removed or requiring increasingly larger incentives to produce the same effect [76]. For example, if accounts were set up for HIV-positive mothers to attend antenatal clinics or payments were made for return visits, would mothers then expect payments for behaviors that previously received no form of compensation? Or at the extreme, would mothers become HIV-positive in order to receive benefits they would not have access to as HIV-negative women? financial incentives are only one behavioral economics lever that are a source of concern. The use of social references through mentor mothers, for example, also raises concern for privacy and loss of social connectedness through the professionalization of mentor mothers [77].

Technology is another challenge. The ability to track couples through the PMTCT protocol can greatly facilitate interventions with underlying behavioral economics concepts, such as text reminders, follow-up from community workers through cell phones and the tracking of patients through the health care system for incentives programs. However, implementation of these high-tech solutions remains a high hurdle even in the most developed parts of the world. Despite these hurdles, organizations are beginning to utilize mobile technology to track and stay in touch with clients through the PMTCT cascade, and more generally to encourage and facilitate a wide range of healthy behaviors [78-81].

While some of these concerns seem daunting, they represent important opportunities that must be addressed in the PMTCT community through rigorous, well-designed studies. The balance of risks and benefits must be considered, piloted and analyzed before incorporating or dismissing the application of behavioral economics in PMTCT.

## Abbreviations

PMTCT: Prevention of mother-to-child transmission; HIV: Human immunodeficiency virus; AIDS: Acquired immunodeficiency syndrome; PCR: Polymerase chain reaction.

## Competing interests

The authors declare that they have no competing interests.

## Authors’ contributions

NKT and AMB conceived of the debate and participated in drafting the manuscript. Both authors read and approved the final manuscript.

## Authors’ information

NKT is a third-year medical student at the Perelman School of Medicine, University of Pennsylvania.

AMB is an assistant professor of nursing at the University of Pennsylvania School of Nursing.

## Pre-publication history

The pre-publication history for this paper can be accessed here:

http://www.biomedcentral.com/1472-6963/13/406/prepub
